# Surveillance of COVID-19 in the General Population Using an Online Questionnaire: Report From 18,161 Respondents in China

**DOI:** 10.2196/18576

**Published:** 2020-04-27

**Authors:** Hongxing Luo, Yongchan Lie, Frits W Prinzen

**Affiliations:** 1 Department of Physiology, Cardiovascular Research Institute Maastricht Maastricht University Maastricht Netherlands; 2 Department of Health Ethics and Society Faculty of Health Medicine and Life Sciences Maastricht University Maastricht Netherlands

**Keywords:** coronavirus, surveillance, syndromic surveillance, participatory surveillance, online questionnaire, Wuhan, COVID-19

## Abstract

**Background:**

The recent outbreak of the coronavirus disease (COVID-19) has become an international pandemic. So far, little is known about the role of an internet approach in COVID-19 participatory surveillance.

**Objective:**

The aim of this study is to investigate whether an online survey can provide population-level information for observing prevalence trends during the early phase of an outbreak and identifying potential risk factors of COVID-19 infection.

**Methods:**

A 10-item online questionnaire was developed according to medical guidelines and relevant publications. It was distributed between January 24 and February 17, 2020. The characteristics of respondents and temporal changes of various questionnaire-derived indicators were analyzed.

**Results:**

A total of 18,161 questionnaires were returned, including 6.45% (n=1171) from Wuhan City. Geographical distributions of the respondents were consistent with the population per province (*R*^2^=0.61, *P*<.001). History of contact significantly decreased with time, both outside Wuhan City (*R*^2^=0.35, *P*=.002) and outside Hubei Province (*R*^2^=0.42, *P*<.001). The percentage of respondents reporting a fever peaked around February 8 (*R*^2^=0.57, *P*<.001) and increased with a history of contact in the areas outside Wuhan City (risk ratio 1.31, 95% CI 1.13-1.52, *P*<.001). Male sex, advanced age, and lung diseases were associated with a higher risk of fever in the general population with a history of contact.

**Conclusions:**

This study shows the usefulness of an online questionnaire for the surveillance of outbreaks like COVID-19 by providing information about trends of the disease and aiding the identification of potential risk factors.

## Introduction

The recent outbreak of the coronavirus disease (COVID-19) has caused over 752,000 confirmed cases and 36,000 deaths as of March 30, 2020 [1-4]. Despite a proactive policy of identifying and treating patients with infected symptoms, it remains resource intensive to screen the general population that is at risk for infection [5,6]. Moreover, inequality of health care systems among different areas brings challenges to cover remote areas, which are also at risk of the COVID-19 infection. Therefore, a new way of surveilling the general population could contribute to our understanding of COVID-19 [7]. The wide use of the internet throughout China, and in the rest of the world, may be sufficient to provide such information. Participatory disease surveillance has been increasingly investigated in recent years as a promising tool to complement traditional facility-based surveillance platforms [8]. It has the advantage of providing quick coverage of a large population during a disease outbreak. Therefore, an online survey may be valuable in monitoring disease trends in communities and providing information for making policies.

In this paper, we report the results of the first online questionnaire about COVID-19, released on January 24 and with data collected up to February 17, 2020. Our study aims to investigate how a history of contact and fever (both defined according to relevant medical guidelines) have evolved during the early phase of government lockdown policies and whether an online questionnaire can be used to identify certain risk factors related to fever among those reporting history of contact.

## Methods

### Questionnaire Development and Distribution

The first version of the questionnaire was developed on January 24, 2020. By that time, little evidence was known about COVID-19. Our anonymous questionnaire was primarily developed from the following 3 sources: (1) the Diagnosis and Treatments of COVID-19 (Third Version) guideline; (2) clinical courses of the first 17 death cases, both of which were released by the National Health Commission of China; and (3) the article that first analyzed the clinical features of 41 cases of COVID-19 [9-11]. The guideline requires a suspected case to satisfy the following criteria: any history of contact including living in Wuhan or having travelled to Wuhan within 2 weeks of disease onset, being in contact with any person with a fever and respiratory symptoms from Wuhan within 2 weeks of disease onset, or belonging to a cluster of infected cases; and clinical manifestations including a fever (defined as a body temperature ≥37.3 °C [99.1 °F]), imaging evidence of COVID-19, normal white blood cell count, or leukopenia or lymphopenia. A confirmed case is further established by positive findings of real time polymerase chain reaction or viral gene sequencing. The descriptions of the guideline are in good consistency with the clinical features of the first 17 death cases and later 41 infected cases reported on January 24, 2020 [9,10]. Therefore, our questionnaire evaluated the risk of COVID-19 in the general population from the following aspects:

1. History of contact: living in Wuhan, having travelled to Wuhan in the past 2 weeks, having any close contact (lived, studied, or worked together, or had any other close contact) in the past 2 weeks with a person with a fever and cough who came from Wuhan, or being in a workplace, school, or family that has at least 2 confirmed cases. Other history of contact with wildlife animals within 2 weeks of disease onset was also considered.
2. Body temperature: having a fever with a body temperature higher than 37.3 °C (99.1 °F)
3. Symptoms: we classified symptoms by their relative importance into the following 3 groups: (1) chief symptoms related to pulmonary infection (ie, cough without sputum or with little sputum) and shortness of breath; (2) secondary symptoms related to systemic changes probably caused by viral infection (ie, fatigue, headache, and myalgia); and (3) probably unrelated symptoms (ie, nasal obstruction, rhinorrhea, sneezing, sore throat, and diarrhea).
4. Comorbidities: Lung diseases, cardiovascular diseases, hypertension, diabetes, stroke, and chronic kidney dysfunction
5. Basic information: age and gender

We did not include laboratory examinations (eg, real time polymerase chain reaction, lymphopenia, white blood cell count) or thoracic imaging results (eg, multiple patchy consolidation and interstitial changes) in our questionnaire because, in general, these would unlikely be obtained by the general population.

By February 17, 2020, we had developed and released three versions of the Chinese questionnaires to the public. They were essentially similar, with the following three major revisions:

1. We divided the age group of ≤40 years used in the first version into age groups of ≤30 years and 31-40 years in the following two versions for better risk stratification.
2. History of contact with wildlife animals was removed from the third version, as we considered it to have a low value for diagnosis in the general population.
3. The question initially included for evaluating shortness of breath, “I feel extremely short of breath when climbing upstairs or walking at a fast speed” (modified from the Medical Research Council Breathlessness scale), was removed from the third version and added as an item named “shortness of breath” to the question about symptoms of COVID-19. This was done because we found an exceptionally high percentage of respondents reporting shortness of breath in the first 2 versions of the questionnaires (26.5% and 32.9%, respectively).

After completing the questionnaire, the respondents would be classified into one of the following 4 risk groups and given different suggestions:

1. High-risk group having history of contact and fever: it was suggested that they measure their body temperature after 30 minutes and immediately visit the hospital to screen for a potential COVID-19 infection.
2. Moderate-risk group having history of contact but without fever: it was suggested that they monitor their body temperature daily and get screened for a potential COVID-19 infection if fever or respiratory symptoms occurred.
3. Low-risk group without history of contact but with fever: this group probably had a common cold, and it was suggested that they make an appointment with a general practitioner for help, if necessary.
4. Very low-risk group without history of contact or fever: they were unlikely to have COVID-19 at the time they completed the questionnaire, and it was suggested that they take necessary measures such as putting on a facemask to prevent the infection.

The questionnaire was developed using a professional online questionnaire website Wenjuanxing (Questionnaire Star) [12]. It is the most popular website for online surveys in China with over 4.2 billion questionnaires recycled and over 59 million users as of February 21, 2020. Questionnaires were distributed online by WeChat (the most popular instant messaging app in China) and sharing the link of the questionnaire. Since our aim was to have an overview of situations in China during the COVID-19 outbreak, we did not target any specific groups of respondents. Distribution and filling out the questionnaires were voluntary, making our study a convenience sampling study.

According to the World Health Organization Guidelines on Ethical Issues in Public Health Surveillance, a surveillance study in emergency outbreak situations is exempted from ethical review and oversight [13]. Indeed, our online questionnaire was designed on January 23, 2020, when the lockdown of Wuhan City was officially announced and released on January 24, so it could not await the formal approval of an ethical review committee. All users were informed at the beginning of the questionnaire that their questionnaire data would be used only for medical education and research purposes. If the informed consent was rejected by the users, they could still continue the questionnaire and obtain their results.

### Data Collection

The questionnaire was released on January 24, 2020, and recycled on February 17. All questionnaire results were downloaded from the website for our analysis. In addition to the items of the questionnaire, the downloaded data also included the date of submission for all respondents as well as the respondents’ location at the city level.

We also collected population data of each province from China Statistical Abstract 2019 published by the National Bureau of Statistics of China [14]. The number of confirmed cases was followed up with on a daily basis since the release of the questionnaire using the NetEase News website, the largest Chinese hub for real time collection of data and news related to COVID-19 [15]. The statistics of confirmed cases per province used in this study were collected until midnight of February 11; at that time, clinically diagnosed cases without positive real time polymerase chain reaction results were also included in the official confirmed number of cases.

### Statistical Analysis

Count data were expressed as number (percentage). Skewed continuous data (time to complete questionnaire) were expressed as median (IQR). Geographical distributions were drawn using Microsoft Excel Visual Basic. A Pearson correlation analysis was used to analyze the relationship between two variables of interest (mainly between date and percentage of respondents of interest per day). Comparison of respondents’ basic characteristics between the inside and outside of Wuhan was performed using a chi-square test or a Fisher exact test if the sample size was <40. Risk of fever in respondents with history of contact was evaluated using a risk ratio (RR) and 95% CI. All statistical analyses were performed using Stata 14.0 (StataCorp) and MATLAB R2018b (MathWorks). Statistical significance was defined as a two-tailed *P* value <*.*05.

## Results

### Questionnaire Respondents

By February 17, 2020 at 2:33 AM, a total of 19,449 individuals completed the questionnaires, 98.02% (n=19,064) from China. After removing 385 questionnaires from overseas countries, 575 lacking informed consent, 55 missing age, 31 missing temperature, 38 missing comorbidities, and 4 missing symptoms information, 18,161 anonymous questionnaires were analyzed. Overall, it took median 52 (IQR 41-67) seconds to complete the questionnaire. Most questionnaires were accessed by clicking on the link of the questionnaire (n=11,337, 62.42%) and by visiting the WeChat mini-app (n=6800, 37.44%).

### Geographical Distributions

**Figure 1:**
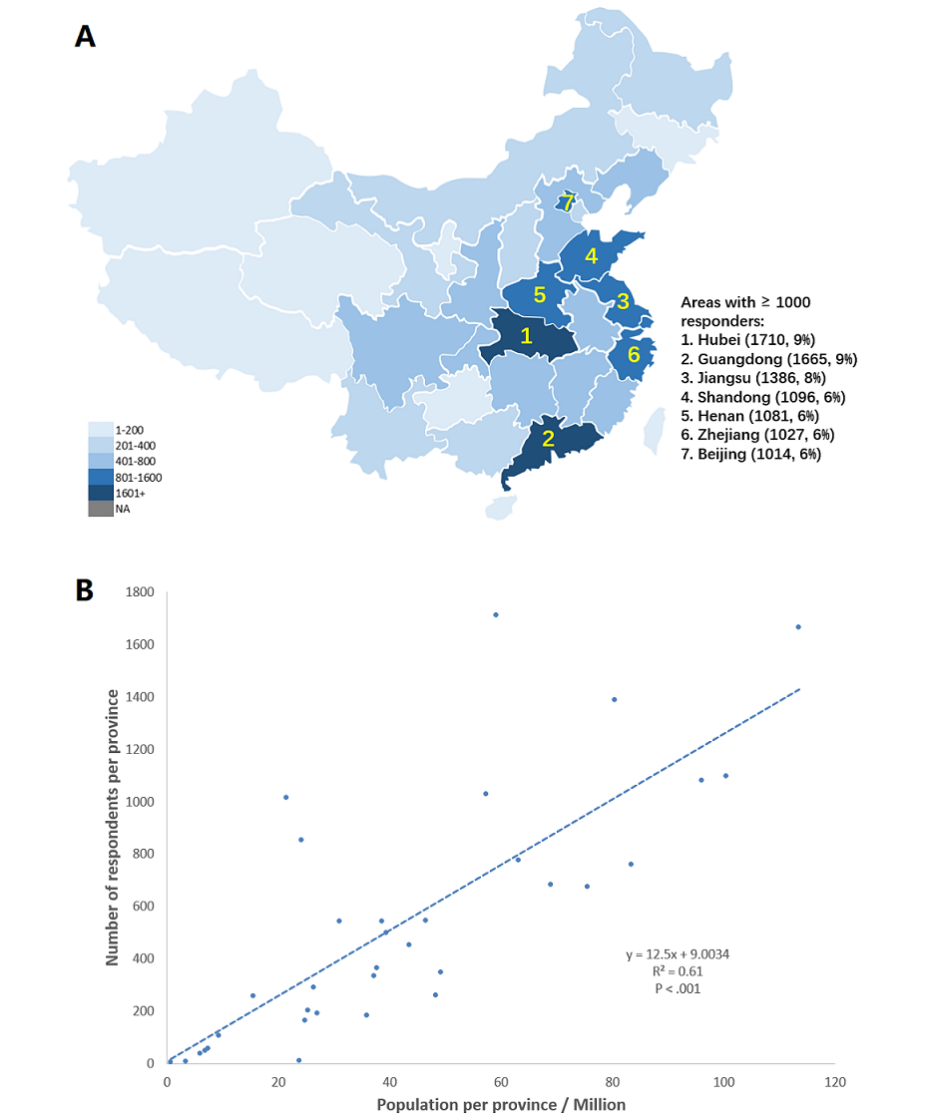
A) Geographical distributions of questionnaire respondents in China. B) A positive correlation between the number of respondents and the size of the population of each province.

Figure 1A shows the geographical distributions of the questionnaire respondents in China. The questionnaire covered all 34 province-level administrative regions. For Hubei Province, 68.48% (n=1171/1710) of the respondents came from Wuhan City, which was most affected by COVID-19. A positive relation was found between the number of respondents and the population size per province (Figure 1B), demonstrating good coverage of the questionnaire across China.

### Basic Characteristics

Table 1 summarizes the demographics and basic characteristics of respondents. The population in Wuhan had similar ages and comorbidities compared with those outside of Wuhan. Age was negatively correlated with the number of respondents (*R*^2^=0.95, *P*<*.*001). As expected, history of contact was more frequent among the respondents living in Wuhan. The percentage of fever was significantly lower among respondents inside versus outside Wuhan. Symptoms were reported in a rather high percentage of respondents. When restricting the symptoms to at least one main symptom and one secondary symptom, the number of respondents with symptoms dropped to 12.62% (n=2292/18,161).

**Table 1:** Demographics and basic characteristics of respondents.

Characteristics		All respondents (N=18,161), n (%)		Wuhan (n=1171), n (%)		Outside Wuhan (n=16,990), n (%)		*P* value
Women		10,801 (59.47)		762 (65.07)		10,039 (59.09)		<.001
**Age (years)**								
	≤30	12,504 (68.85)	782 (66.78)		11722 (68.99)		.11	
	31-40	3757 (20.69)	282 (24.08)		3475 (20.45)		.003	
	41-50	1154 (6.35)	70 (5.98)		1084 (6.38)		.59	
	51-60	532 (2.93)	28 (2.39)		504 (2.97)		.26	
	61-70	147 (0.81)	6 (0.51)		141 (0.83)		.24	
	≥71	67 (0.37)	3 (0.26)		64 (0.38)		.51	
**Comorbidity**		1593 (8.77)		95 (8.11)		1498 (8.82)		.41
	Hypertension	655 (3.61)	38 (3.25)		617 (3.63)		.49	
	Lung diseases	468 (2.58)	24 (2.05)		444 (2.61)		.24	
	Cardiovascular diseases	375 (2.06)	21 (1.79)		354 (2.08)		.50	
	Diabetes	223 (1.23)	16 (1.37)		207 (1.22)		.66	
	Chronic kidney disease	135 (0.74)	5 (0.43)		130 (0.77)		.19	
	Stroke	34 (0.19)	4 (0.34)		30 (0.18)		.21	
**History of contact**		2631 (14.49)		1171 (100.00)		1460 (8.59)		<.001
	Living in Wuhan now or having gone to Wuhan in the past 2 weeks	1950 (10.74)	1171 (100.00)		779 (4.59)		<.001	
	Contact with a person with fever and cough from Wuhan in the past 2 weeks	938 (5.16)	298 (25.45)		640 (3.77)		<.001	
	At least 2 confirmed cases in workplace, school, or family	532 (2.93)	122 (10.42)		410 (2.41)		<.001	
**Symptoms**		11,796 (64.95)		699 (59.69)		11,097 (65.31)		<.001
	Fever	1653 (9.10)	56 (4.78)		1597 (9.40)		<.001	
	Cough	5242 (28.86)	314 (26.81)		4928 (29.00)		.11	
	Shortness of breath	4393 (24.19)	263 (22.46)		4130 (24.31)		.15	
	Nasal obstruction, rhinorrhea, or sneezing	4376 (24.10)	237 (20.24)		4139 (24.36)		.001	
	Sore throat	3397 (18.70)	201 (17.16)		3196 (18.81)		.16	
	Fatigue	3245 (17.87)	148 (12.64)		3097 (18.23)		<.001	
	Headache or myalgia	2072 (11.41)	87 (7.43)		1985 (11.68)		<.001	
	Diarrhea	1360 (7.49)	70 (5.98)		1290 (7.59)		.04	

### History of Contact

**Figure 2:**
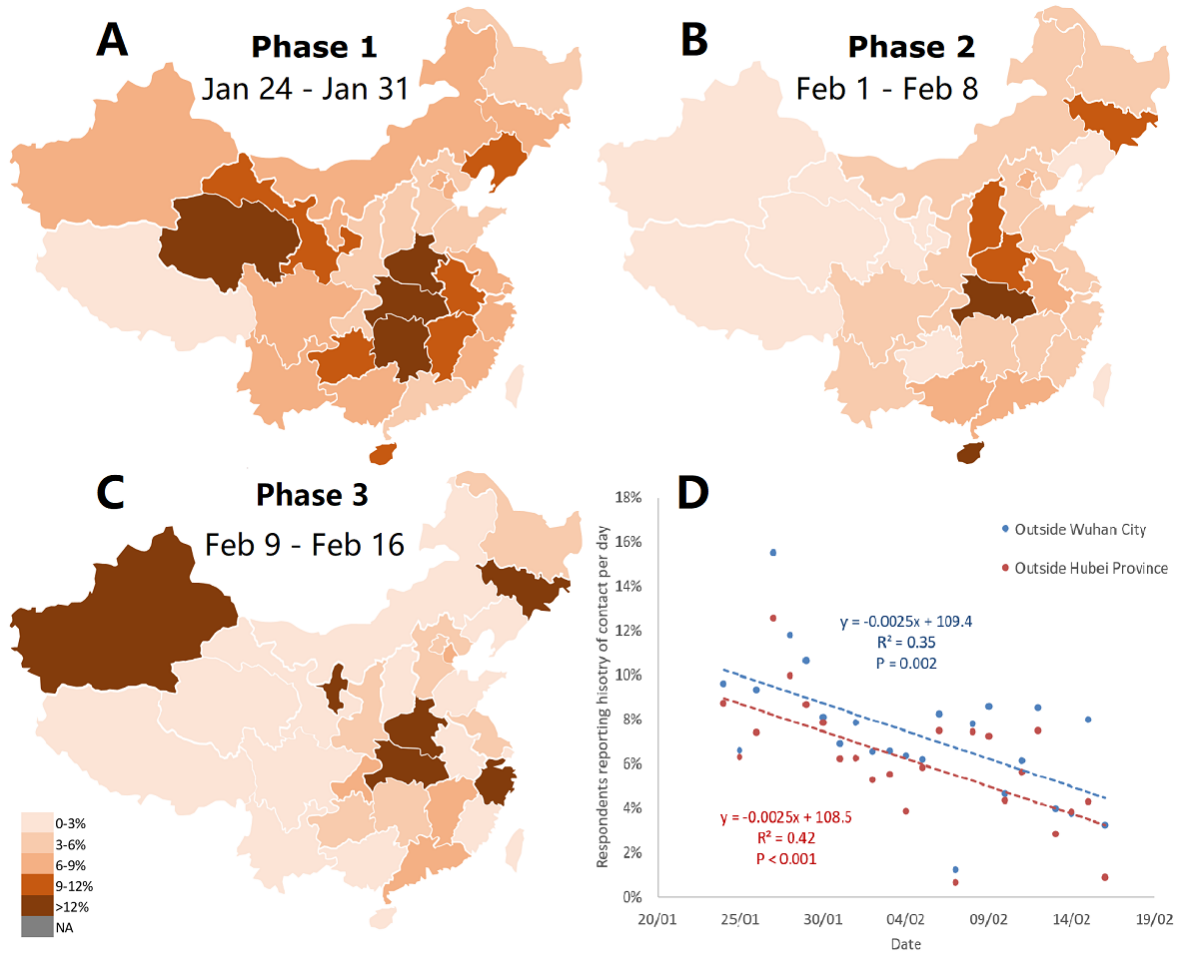
The geographic spread of the proportion of respondents reporting a history of contact in three phases of the COVID-19 outbreak (A, B, and C), and its time course in all regions outside Wuhan City and Hubei Province (D).

A history of contact was reported by more than one-eighth of respondents. However, the high percentage might have been confounded considering that all respondents living in Wuhan City had a history of contact according to the definition of the official guideline, so we excluded these respondents from our analysis and divided the remaining respondents by every 8 days into 3 phases: phase 1 was from January 24 to 31, phase 2 was from February 1 to 8, and phase 3 was from February 9 to 16. Despite heterogeneous responses of different provinces, the proportion of respondents reporting a history of contact had markedly decreased over these 3 phases in most provinces (Figure 2A, B, and C). This observation was further confirmed by correlation analysis between the proportion of respondents reporting a history of contact and date in areas outside of Wuhan City and Hubei Province (Figure 2D). These findings indicate the efficacy of current policies adopted to reduce the history of contact among the general population since the lockdown in Wuhan and other areas on January 23, 2020.

### Body Temperature

**Figure 3:**
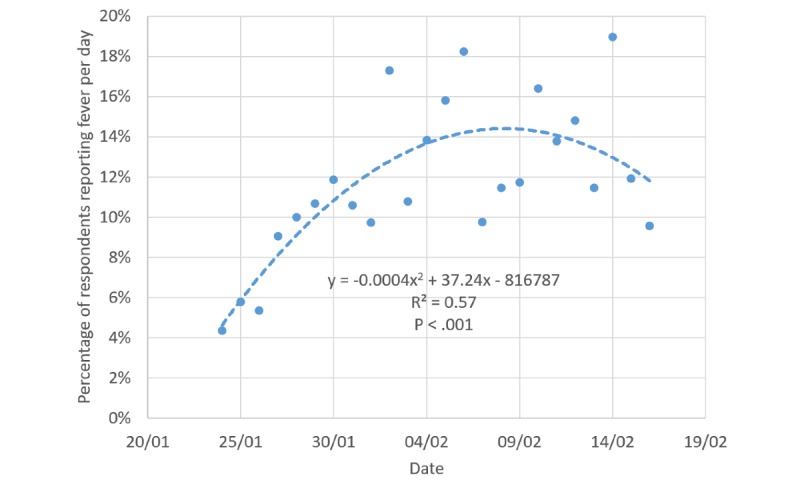
Proportion of respondents reporting a fever over time.

Body temperature was measured in 77.49% (n=14,073/18,161) of respondents, with a higher percentage in Wuhan City (n=990/1171, 84.54%) and Hubei Province (n=1431/1710, 83.68%). Overall, fever was reported in less than one-tenth of the respondents. Unexpectedly, a lower percentage was found for Wuhan City and Hubei Province. This might be due to COVID-19 developing to a further stage in Wuhan, and fever cases were identified early and sent to hospitals without access to the internet. We further analyzed how the percentage of respondents with fever evolved with time. The trend seemed to peak on around February 8, 2020 (Figure 3).

### Fever in Respondents With a History of Contact

Analyzing the relationship between fever and history of contact may help develop population-based strategies for prevention purposes. For the respondents living outside Wuhan, we found a significant relation between any history of contact and fever (RR 1.31, 95% CI 1.13-1.52, *P*<.001). Travelling to Wuhan, having any close contact with a confirmed case, and having at least 2 confirmed cases at the workplace in the past 2 weeks conferred a significantly higher risk of fever (RR 1.47, 95% CI 1.23-1.77, *P*<.001; RR 1.98, 95% CI 1.67-2.24, *P*<.001; and RR 2.12, 95% CI 1.74–2.58, *P*<.001, respectively). Moreover, there was a significant positive relation between the number of officially confirmed cases and the number of respondents reporting a fever (*R*^2^=0.41, *P*<.001) or the number of respondents reporting a fever and a history of contact (*R*^2^=0.35, *P*<.001) on a province basis. Regarding risk stratification based on history of contact and fever, most respondents (n=14,264/18,161, 78.54%) were classified in the very low-risk group, followed by the moderate-risk group (n=1883, 10.37%) and the low-risk group (n=1428, 7.86%), whereas only 1.24% (n=225) were classified to the high-risk group.

**Figure 4:**
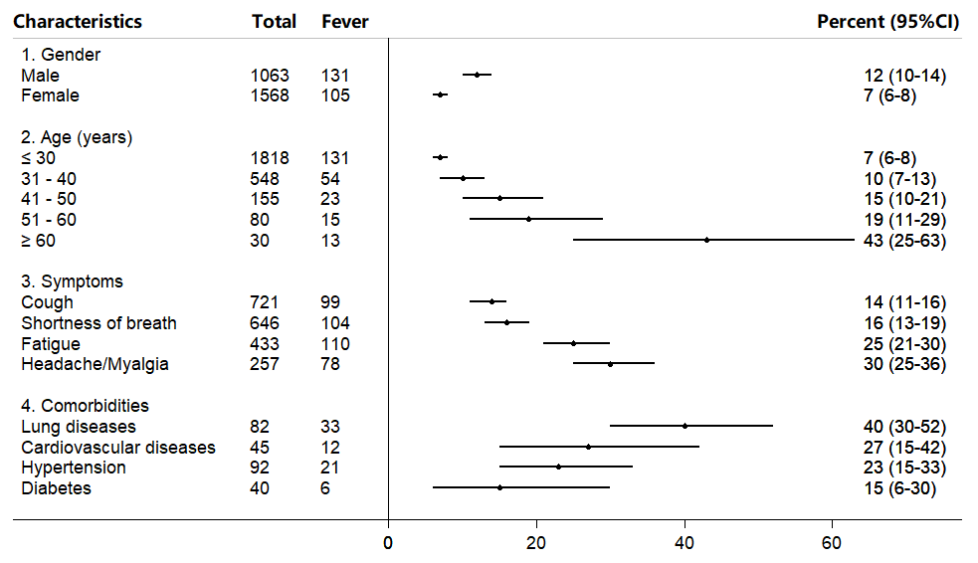
Fever in various subgroups of respondents with history of contact.

Furthermore, comparison of fever rates among groups of various characteristics was likely to help identify risk factors (Figure 4). Males were at a higher risk of fever than females (*P*<.001). There was a positive trend between age and fever (*P*<.001). Respondents reporting fatigue and headache or myalgia were more likely to report fever (*P*<.001). Comorbidities showed various associations with fever, among which history of lung diseases seemed to confer a higher risk of fever than the others. However, the relationship needs to be further validated by studies with larger samples because of a relatively small number of respondents in each group.

## Discussion

### Principal Findings

To the best of our knowledge, this is the first large-sample online surveillance of the COVID-19 outbreak in the general population. Our major findings include: the questionnaire had a good coverage of all provinces of China in a relatively short period of time (about 3 weeks); the history of contact among the population outside of Wuhan and Hubei Province significantly decreased during the early phase of the government lockdown policy; fever reported by respondents significantly increased in the short-term of the disease outbreak and levelled off in 2-3 weeks; and, among those with history of contact, some factors (male, advanced age, and history of lung diseases) seemed to be associated with a higher risk of fever.

### Values of Online Questionnaire

An online questionnaire is likely to serve as a complementary way of disease surveillance in the general population, especially during the emergent outbreak of an infectious disease [5]. It takes the advantage of low costs and efficient delivery to all areas, even the most remote areas where internet access is better than health care resources [16,17]. Our questionnaire was completed by 385 Chinese respondents from 38 overseas countries, including developed (the United States, Japan, Canada, and the United Kingdom), developing (Brazil, Russia, India, and South Africa), and underdeveloped countries (Laos, Uganda, and Cambodia). Translation of the questionnaire to other languages may further increase the coverage across the world and improve surveillance of the COVID-19 outbreak and comparable epidemics.

Compared with the conventional way of disease surveillance, the online questionnaire covers the population with generally less severe conditions but, nevertheless, is at risk of infection [7,18], taking into account that this population helps to establish the full spectrum of COVID-19 epidemiology. It may also facilitate the early triage and diagnosis of high-risk groups when combined with other digital health measures such as online physician consultation, which has been widely adopted since the COVID-19 outbreak in China. For the low-risk population, the questionnaire can also be adapted to reduce unnecessary anxiety and hospital visits, and thus, greatly relieve the workloads of health care facilities, especially when an emergent public health event occurs [19].

The questionnaire approach is advantageous compared with other approaches of online disease surveillance using data from Google Trends, Twitter, or Facebook [20-22]. It provides richer information of the respondents, as most items can be designed according to medical guidelines and characteristics of target populations. Therefore, it is a more active approach than other infosurveillance methods using social media. The information such as symptoms, history of contact, and comorbidities provided by an online questionnaire can be further combined with vital data such as body temperature, heart rate, respiratory rate, oxygenation level, and activity level obtained from wearable devices to have a more comprehensive and reliable estimation of respondent’s risk of disease [23]. For the high-risk group identified using an online questionnaire, a case can be further confirmed by sending a home-testing kit and instructing the respondents to perform a rapid diagnostic test, as shown in the GoViral study [24]. Additionally, self-reported data from an online questionnaire can be linked with electronic medical records to build a long-term monitoring system [8].

### Use of Questionnaire to Observe Trends

An online survey is likely to be used to observe the trends of disease prevalence in communities and, thus, support government policy evaluation. In our study, the date February 8, 2020, when the percentage of fever respondents peaked, was 16 days following the lockdown of Wuhan City, which was close to the 14 days of the maximum incubation period of the coronavirus [25]. The delay of the fever peak might be associated with delayed quarantine policies in other cities in China. Overall, our data supported the efficacy of current policies (quarantine, social distancing, and isolation of infected populations) for containing the spread of COVID-19 from Wuhan City to the other areas of China [6,26,27]. However, the period and efficacy of quarantine may differ by country [28]. It depends on not only government policies but also local culture and more importantly active support from the general population. For other countries, which may not have quarantine policies as strict as China, the time to fever peak is probably longer among the general population. Moreover, integration of the survey data into a model for real time and long-term forecasting of disease trends is likely to provide richer information for making policies [29]. Of note, our questionnaire is more applicable to those living in China than abroad. The definition of history of contact has mostly relied on contact with a confirmed case from Wuhan. However, this can be further modified according to the earliest and generally most severely affected area of a country of interest, such as Lombardy in Italy.

### Use of Questionnaire to Identify Risk Factors

Our survey also indicates that some factors such as male, an advanced age, and a history of lung disease are likely to relate to a higher risk of infection, and thus, these groups should be under close observation. Indeed, these risk factors identified from our study are consistent with the clinical features of infected cases in previous publications [9,30-33]. By quickly disseminating an online questionnaire during the early phase of a disease outbreak, risk factors can be identified at a much earlier phase rather than when enough severe cases have been collected and analyzed using a conventional surveillance method. This further allows for earlier protection of vulnerable groups from potential infection and, thus, reduces the number of cases. Internet-based surveillance approaches based on Twitter have been demonstrated to detect Ebola, avian influenza, and thunderstorm asthma at an early stage, even before the first official report [20-22].

### Limitations of the Approach

The approach undoubtedly has the bias of sampling primarily internet users and their relatives. As a consequence, the population included in our study is relatively young. A previous study demonstrated that both too young (age 0-10 years) and too old (age older than 81 years) populations are underrepresented in an internet-based monitoring survey [34]. A better coverage of the general population with high representativeness generally requires a more complicated study design together with robust supports from an official institution [8]. The questionnaire can also be distributed through other web platforms such as Sina Weibo (the most popular microblogging website in China) and news media (NetEase and Xinhua), which have a wider reach of respondents in China. Furthermore, this study does not include a follow-up for individual patients. This choice was made to respect the respondents’ privacy. However, in future studies it may be acceptable to allot an individual code to each individual, thereby allowing follow-ups; although, systematic follow-ups will remain a problem with internet questionnaires. Follow-ups may be further compromised by the lack of internet access when the individual is hospitalized.

Unlike hospitals, which diagnose COVID-19 using a comprehensive set of laboratory and imaging examinations, we did not include diagnostic tests such as real time polymerase chain reaction or lung computed tomography results in our questionnaire. Therefore, evaluating the respondents’ risk of viral infection from the history of contact, body temperature, symptoms, and comorbidities may have the risk of underestimating some patients who are asymptomatic or presymptomatic, which are not uncommon [35,36].

Based on this study, we have updated our fourth version of the Chinese questionnaire [37] and released the English questionnaire [38] (also see Multimedia Appendices 1 and 2 for Word format files). Both questionnaires follow the Attribution 4.0 International license, meaning that they are free to be shared and adapted under the condition that this work has been properly cited. Considering privacy purposes, the survey data of this study can be obtained from the corresponding author at request.

### Conclusions

This study shows that an online questionnaire may help monitor current prevalence, evaluate government policy, and identify high-risk populations during the COVID-19 outbreak. The online questionnaire approach can also be adapted to monitor other types of infectious diseases depending on areas of interest.

## Appendix

Multimedia Appendix 1 Coronavirus Infection Risk Self-Assessment Questionnaire (CIRSAQ 4.0).

Multimedia Appendix 2 Coronavirus Infection Risk Self-Assessment Questionnaire (CIRSAQ 4.0) - Chinese Simplified.

## Abbreviations

COVID-19: coronavirus disease
RR: risk ratio
