# Supplementary material for: Surveillance of COVID-19 in the General Population Using an Online Questionnaire: Report From 18,161 Respondents in China
Source: JMIR Public Health Surveill. 2020 Apr 27;6(2):e18576. doi: 10.2196/18576 (PMC7187763; doi:10.2196/18576)
Supplement: Multimedia Appendix 1 [file publichealth_v6i2e18576_app1.docx]

**Coronavirus Infection Risk Self-Assessment Questionnaire (CIRSAQ 4.0)**

1. This CIRSAQ 4.0 is based on the Prevention and Treatments of 2019 Coronavirus Disease Guideline (Fifth Version) released by the National Health Commission of China on 21 February 2020 and relevant medical papers. The results provided are for personal reference only and cannot replace the doctor's diagnosis. This tool is used anonymously and data would be used for research and teaching purposes only. *

| Agree |
| --- |
| Not agree |

2. I am living in Wuhan now, or travelled to Wuhan in the past 2 weeks. *

| Yes |
| --- |
| No |

3. In the past 2 weeks, I lived, studied or worked together, or had any other close contact with a person with fever and cough who came from Wuhan. *

| Yes |
| --- |
| No |

4. My workplace, school or family has at least 2 confirmed cases. *

| Yes |
| --- |
| No |

5. I am having a fever with body temperature higher than 37.3 ℃ (99.1℉) *

| Yes |
| --- |
| No |
| I have not measured yet. |

6. Now I feel (single or multiple choices) *

| Fatigue |
| --- |
| Cough without sputum or with little sputum |
| Shortness of breath |
| Headache or myalgia |
| Nasal obstruction, rhinorrhea, sneezing |
| Sore throat |
| Diarrhea |
| None |

7. I have the following diseases (single or multiple choices) *

| Hypertension |
| --- |
| Lung disease |
| Heart disease |
| Diabetes |
| Chronic kidney dysfunction |
| Stroke |
| None |

8. My age is (years) *

| ≤ 30 |
| --- |
| 31-40 |
| 41-50 |
| 51-60 |
| 61-70 |
| ≥ 71 |

9. My gender is *

| Man |
| --- |
| Woman |

10. I am a doctor or nurse *

| Yes |
| --- |
| No |
