# Supplementary material for: Surveillance of COVID-19 in the General Population Using an Online Questionnaire: Report From 18,161 Respondents in China
Source: JMIR Public Health Surveill. 2020 Apr 27;6(2):e18576. doi: 10.2196/18576 (PMC7187763; doi:10.2196/18576)
Supplement: Multimedia Appendix 2 [file publichealth_v6i2e18576_app2.docx]

**肺炎风险自我评估（第四版）**

1. 根据国家卫生健康委2020年2月21日最新发布的《新型冠状病毒肺炎防控方案（第五版）》及相关医学论文编制而成。结果仅供个人参考，不能替代专业医生的诊断。本评估工具为匿名回答，数据仅用于科研教学。 *

| 同意使用 |
| --- |
| 不同意 |

2. 我现在居住在武汉，或最近2周去过武汉 *

| 是的 |
| --- |
| 没有 |

3. 最近2周，我接触过（共同居住、学习、工作或有其他密切接触）来自武汉的发热及咳嗽的人 *

| 是的 |
| --- |
| 没有 |

4. 我的工作单位、学校或家庭至少有2例确诊病例 *

| 是的 |
| --- |
| 没有 |

5. 我正在发热，体温高于37.3摄氏度（单选） *

| 是的 |
| --- |
| 没有 |
| 没测体温，不知道 |

6. 我现在感觉（可多选） *

| 疲劳、全身乏力 |
| --- |
| 咳嗽、但没有痰，或有少量痰 |
| 喘气，或呼吸困难 |
| 头痛、全身肌肉酸痛 |
| 鼻塞、流鼻涕、打喷嚏 |
| 喉咙痛 |
| 腹泻 |
| 没有上述症状 |

7. 我有以下疾病 （可多选） *

| 高血压 |
| --- |
| 肺部疾病 |
| 心脏病 |
| 糖尿病 |
| 肾功能不全 |
| 脑中风 |
| 都没有 |

8. 我的年龄（岁，单选） *

| ≤ 30 |
| --- |
| 31-40 |
| 41-50 |
| 51-60 |
| 61-70 |
| ≥ 71 |

9. 我的性别 *

| 男 |
| --- |
| 女 |

10. 我是医生或护士 *

| 是 |
| --- |
| 否 |
